# Retraction Note: Digital transformation, green innovation, and carbon emission reduction performance of energy-intensive enterprises

**DOI:** 10.1038/s41598-024-57674-y

**Published:** 2024-03-27

**Authors:** Xiao-meng Liu, Ying-quan Zhang

**Affiliations:** 1 Business School, Jinling Institute of Technology, Nanjing, 211169 Jiangsu People’s Republic of China; 2 Business School, Hohai University, Nanjing, 211100 Jiangsu People’s Republic of China

Retraction of: *Scientific Reports* 10.1038/s41598-024-54587-8, published online 16 February 2024

The Authors have retracted this Article.

After publication, the Authors discovered that the data underlying the analysis are inaccurate. Corporate sustainability reports were scored based on a combination of non-disclosure/disclosure and qualitative/quantitative information on a range from zero to two, with the final value being the sum of the scores of all indicators. During follow-up investigations, the Authors discovered that a substantial number of corporate sustainability reporting disclosures of the studied enterprises contained incomplete data.

The Authors therefore no longer have confidence in the robustness of their analysis and conclusions.

Both Authors agree with this retraction.

